# Investigation of Biochemical Alterations in Ischemic Stroke Using Fourier Transform Infrared Imaging Spectroscopy—A Preliminary Study

**DOI:** 10.3390/brainsci9110293

**Published:** 2019-10-25

**Authors:** Fazle Rakib, Carmen M. Ali, Mohammed Yousuf, Mohammed Afifi, Pooja R. Bhatt, Ehsan Ullah, Khalid Al-Saad, Mohamed H. M. Ali

**Affiliations:** 1 Department of Chemistry and Earth Sciences, Qatar University, Doha 2713, Qatar; fazle@qu.edu.qa (F.R.); carmen@qu.edu.qa (C.M.A.); m_afifi_h2o@hotmail.com (M.A.); poojarbhatt1@gmail.com (P.R.B.); 2 Central Laboratory Unit (CLU), Qatar University, Doha 2713, Qatar; mdyousuf@qu.edu.qa; 3 Qatar Computing Research Institute (QCRI), Hamad Bin Khalifa University (HBKU), Qatar Foundation (QF), Education City, Doha 34110, Qatar; eullah@hbku.edu.qa; 4 Diabetes Research Center, Qatar Biomedical Research Institute (QBRI), Hamad Bin Khalifa University (HBKU), Qatar Foundation (QF), Doha 34110, Qatar; 5 Qatar National Library, Doha 5825, Qatar

**Keywords:** Fourier transform infrared (FTIR) imaging spectroscopy, transmission electron microscope (TEM), brain ischemia, photothrombotic stroke

## Abstract

Objective: Brain damage, long-term disability and death are the dreadful consequences of ischemic stroke. It causes imbalance in the biochemical constituents that distorts the brain dynamics. Understanding the sub-cellular alterations associated with the stroke will contribute to deeper molecular understanding of brain plasticity and recovery. Current routine approaches examining lipid and protein biochemical changes post stoke can be difficult. Fourier Transform Infrared (FTIR) imaging spectroscopy can play a vital role in detecting these molecular alterations on a sub-cellular level due to its high spatial resolution, accuracy and sensitivity. This study investigates the biochemical and molecular changes in peri-infract zone (PIZ) (contiguous area not completely damaged by stroke) and ipsi-lesional white matter (WM) (right below the stroke and PIZ regions) nine weeks post photothrombotic ischemic stroke in rats. Materials and Methods: FTIR imaging spectroscopy and transmission electron microscopy (TEM) techniques were applied to investigate brain tissue samples while hematoxylin and eosin (H&E) stained images of adjacent sections were prepared for comparison and examination the morphological changes post stroke. Results: TEM results revealed shearing of myelin sheaths and loss of cell membrane, structure and integrity after ischemic stroke. FTIR results showed that ipsi-lesional PIZ and WM experienced reduction in total protein and total lipid content compared to contra-lesional hemisphere. The lipid/protein ratio reduced in PIZ and adjacent WM indicated lipid peroxidation, which results in lipid chain fragmentation and an increase in olefinic content. Protein structural change is observed in PIZ due to the shift from random coli and α-helical structures to β-sheet conformation. Conclusion: FTIR imaging bio-spectroscopy provide novel biochemical information at sub-cellular levels that be difficult to be obtained by routine approaches. The results suggest that successful therapeutic strategy that is based on administration of anti-oxidant therapy, which could reduce and prevent neurotoxicity by scavenging the lipid peroxidation products. This approach will mitigate tissue damage in chronic ischemic period. FTIR imaging bio-spectroscopy can be used as a powerful tool and offer new approach in stroke and neurodegenerative diseases research.

## 1. Introduction

Brain stroke is a highly complex mechanism and involves a broad range of hemodynamic and molecular interventions [1,2]. Disruption of blood circulation due to stroke is detrimental for the brain function due to its high demand for oxygen and glucose. As a result, accumulation of toxic metabolites and energy deficit within affected cells takes place by vascular occlusion [3]. Stroke influences excitotoxicity [4], production of reactive free radicals [5,6], formation of tissue acidosis [7], inflammation and programmed cell death (apoptosis) as well as peri-infarct depolarization [3,8]. Contiguous regions of stroke affected tissue undergo morphological, cellular and sub-cellular changes, such as gliosis [9], lipid peroxidation [10,11] and protein malformation [5]. Previous studies show that neuronal networks reorganize after stroke and such lost functions are regained [12,13]. 

Conventional technique, such as magnetic resonance imaging (MRI) and Diffusive tensor imaging (DTI) [14,15,16] have been applied in vivo imaging of brain tissues to characterize the biochemical and anatomical alterations post-stroke [17,18,19]. Although these techniques provide important data, they yield spatial resolution of about 250 µm which is not sufficient to study biochemical and biomolecular changes at cellular and sub-cellular levels. Routine histology [14,18,19] and immunohistochemistry (IHC) [14] also have been used to examine the biochemical and morphological changes in the brain tissues [5,17,18,19]. Whereas, histology and IHC give high spatial resolution they are disrupting the tissue morphology and only limited to certain biochemical markers. In order to provide detailed information about these sub-cellular changes higher spatial resolution imaging technology and is needed.

Fourier Transform Infrared (FTIR) imaging spectroscopy is able to screen changes within of biological samples on a molecular level [5,20,21]. By means of FTIR imaging, one should be able to observe molecular changes of brain tissues and their spatial distribution [5,22]. The elegance of this technique is that it needs almost no sample preparation other than sectioning as per normal tissue techniques, leaving the sample unchanged after collection of the IR spectra. This assures reproducible measurements and makes FTIR convenient for combining with other standard staining techniques and other molecular probes [5,20,23]. FTIR imaging has the ability to detect, simultaneously, discrete changes in molecular structure and composition of tissues, which cannot be done by standard histological staining methods. Single data acquisition can provide direct biochemical analyses of all macromolecular components within tissue samples without the addition of chemical stains or reagents and without disrupting the tissue morphology [24,25]

Changes of neuronal structure due to neurodegenerative diseases such as Alzheimer’s [26] or Parkinson [25] and neurological infection, that is, cerebral malaria [27] have successfully been investigated by means of FTIR spectroscopy [28,29]. In the study of Choo et al. [30,31] it was shown that FTIR spectroscopy can determine Alzheimer’s disease and classify white and grey matter of brain with their improvement compared with classical pathological method. Possible mechanisms of functional repair after stroke are associated with neuronal modification and biochemical alterations [32,33,34]. Caine et al. showed the possibility of FTIR application in recognizing biochemical changes due to mechanical affect in 24 h. post stroke brains [22]. Mark et al. demonstrated application of FTIR spectroscopy for simultaneous direct spectroscopic imaging of brain glycogen and lactate, in situ within ex vivo tissue sections in 1 h, 1 day, 2 days and 3 days post stroke [35]. Ali et al. investigated the bio-molecular, elemental and chemical effect caused by 1-week post stroke distorting the integrity, structure and functionality of the brain by FTIR and laser ablation (LA) [5]. Ali et al. also showed the sensitivity and high resolution of FTIR spectroscopy in characterizing different brain tissue regions [23]. By choosing optimal biomarkers, FTIR not only discriminates diseased from healthy tissue but it also is capable of grading stages of malignancy, which is very important for determining treatment methods [20,36,37]. Richter et al. have combined FTIR measurements with positron emitting tomography (PET) in order to identify tumor tissues [38].

In this paper, we present FTIR spectral and imaging analysis of the contra- and ipsi-lesional regions (cortex and white matter (WM)) of nine weeks post stroke in rats brain tissues. We also correlated changes in the tissue’s ultrastructure (by transmission electron microscopy (TEM)) and biochemical alterations (by FTIR) in order to assess the best therapeutic approach. In this study, we have used the photothrombotic stroke model in order to study and analyze the cellular and molecular changes due to well defined location, minimal surgical intervention and long-term study of impairment and recovery [39,40]. Studies found out partial recovery towards chronic stage at 9 weeks post stroke and this recovery is combined with an increase in the functional connectivity of the contra-lesional and ipsi-lesional hemisphere. This is believed to be associated with reorganization of surviving neural networks which provides opportunities for therapies that target intrinsic neurorestorative mechanisms related to recovery [41,42]. Consequently, the aim of this research work is to makes advances in stroke bio-diagnostics during this time point, which may improve treatment and provide links between tissue injury and repair.

## 2. Materials and Methods

### 2.1. Animal Model

Six male Sprague-Dawley rats (11-week-old, Charles River Laboratories International, Wilmington, MA, USA) subjected to photothrombotic stroke (n_stroke_ = 6), which was induced (illuminating the brain through intact skull with a cold-light source after anesthetized) in the right sensorimotor cortex. Nine weeks post stroke induction animals were sacrificed with an overdose of isoflurane followed by transcranial perfusion. The experimental animals protocol was approved by the by the University of Utrecht Animal Research Ethics Board and adhered to the Netherlands Council on Animal Care guidelines for humane animal use [5,23].

### 2.2. Block and Tissue Preparation

Stroke brains were removed, fixed in 4% paraformaldehyde (PFA) for 24 hours and embedded in paraffin blocks. Paraffin embedded tissue blocks were serially cut into 10 μm slices using Leica RM 2155 semi-automated rotary microtome (Leica Microsystems Nussloch GmbH, Nussloch, Germany). For the stroke-affected brains, thin coronal sections of brain were taken from the central location of the lesion (Bregma 1.20 mm). Tissue preparation and routine histology and was performed on whole brain tissue to observe contra-lesional and ipsi-lesional hemispheres tissue morphology as described in our previous study [5,23]. The regions of interest (ROI) for this study are: a) peri-infract zone (PIZ) (area surrounding the stroke region, part of gray matter) and b) ipsi-lesional white matter (WM) (area adjacent and right below to PIZ).

### 2.3. Transmission Electron Microscopy

Brain tissue was collected from the experimental animals and diced into 1 mm square pieces immersed in 2% buffered glutaraldehyde and subsequently fixed in the same fixative to be stored/transported and held in a fridge at 4 °C. The control healthy and stroke affected brain regions were conventionally fixed, rinsed, dehydrated using series of graded acetone and phosphate buffer and then impregnated with fresh resin (Agar Scientific 100 resin kit). Brain sections were cut using an RMC ultra-microtome at a thickness below 100 nm (70 nm) then stained using uranyl acetate and lead citrate. The samples were examined using a FEI TF20 TEM (FEI, Brno-Černovice, Czech Republic) at an acceleration voltage of 100 kV. The Photo-micro-graphs were digitally acquired by a 4 × 4 BM-Eagle digital camera (FEI, Brno-Černovice, Czech Republic).

### 2.4. FTIR Data Acquisition and Pre-Processing

The brain sections for FTIR analysis for the stroke animal model were mounted on a BaF_2_ window (Korth Kristalle GmbH, Altenholz, Germany), deparaffinized, dehydrated, dried out and stored at room temperature in a desiccator. FTIR spectra were recorded using an Agilent FTIR Cary 620 micro-spectrometer within the spectral range of 4000–700 cm^−1^ with 64 scans per spectrum, 4 cm^−1^ spectral resolution and spatial resolution 80 μm. FTIR chemical images were recorded using a 64 × 64 Mercury Cadmium Telluride (MCT) Focal Plane Array (FPA) liquid nitrogen cooled detector in mosaic mode. FTIR imaging was performed with a pixel size of 5.5 × 5.5 µm. Resolution Pro. Software (version 5.0) (Agilent Technologies, Santa Clara, CA, USA), Cytospec (version 2.00.06) (Cytospec, Berlin, Germany), MATLAB (version 2018b) (The Mathworks, Natick, MA, USA) and OriginPro (2019) (OriginLab Corporation, Northampton, MA, USA) are used for image generation and pre-processing of all spectral data. Spectra were acquired away from the border between tissue regions and the substrate to avoid resonance Mie scattering. Detailed FTIR data acquisition and pre-processing of the data are explained in our previous work [5,20,23]. The spectra from contra-lesional (GM and WM) and ipsi-lesional (PIZ and WM), were collected, averaged and then compared in this study. Detailed FTIR band assignments were described in Table 1.

### 2.5. Statistical Analysis

A paired t-test was performed with Origin Lab. 2019 to assess significant differences between the molecular contents of the cortex and WM tissues of contra-lesional and ipsi-lesional regions (PIZ and WM) of nine weeks post stroke brains. Mean and standard deviation was generated and individual animal values were averaged for each group. Mann Whitney-U test was performed on all data to test significant differences (*p* < 0.05). All t-tests were two tailed and the p values less than or equal to 0.05 were considered statistically significant for comparisons * *p* ≤ 0.05; ** *p* ≤ 0.001 with a 95% confidence interval.

## 3. Results

**Figure 1:**
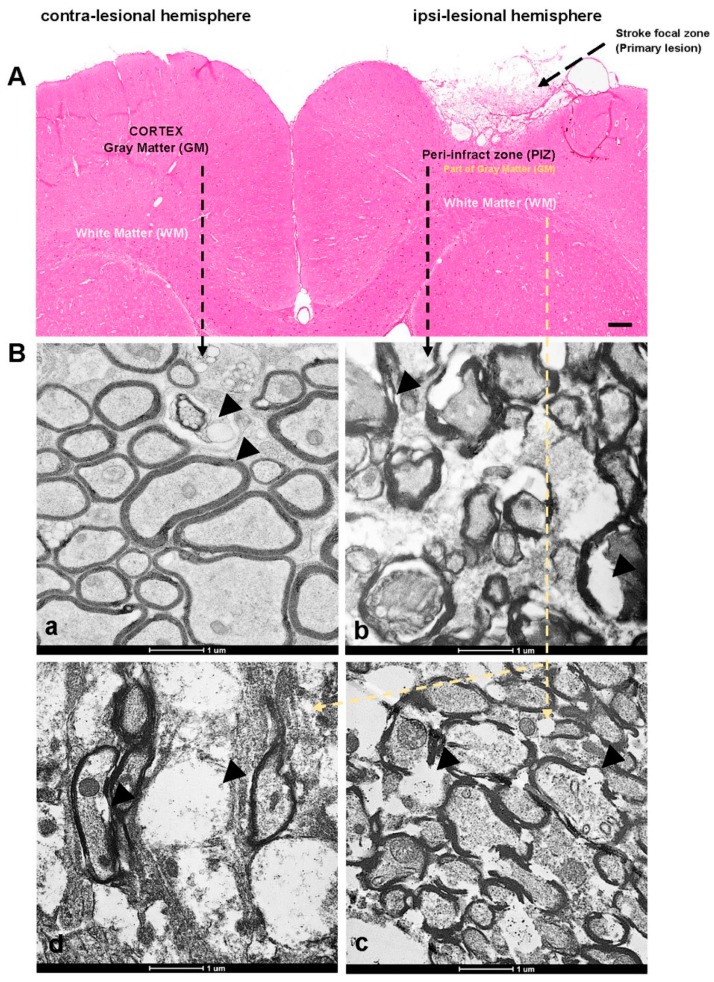
The hematoxylin and eosin (H&E) staining and transmission electron microscopy (TEM) images. (**A**) Contra-lesional and Ipsi-lesional hemispheres of nine weeks stroke brain tissues showing the regions of interest—peri-infract zone (PIZ) and white matter (WM). (**B_a_**) Representative TEM images of contra-lesional cortex (unaffected neurons), whereas (**B_b_**) representing ipsi-lesional PIZ (affected side) and (**B_c–d_**) representing ipsi-lesional WM showing the ultrastructural changes—axonal shearing, formation of vacuoles and swelling marked with black arrows on the images. Scale bar = 100 µm for H&E and 1 µm for TEM.

The nine weeks post photothrombotic ischemic strokes were confirmed by histology (H&E staining Figure 1A). The H&E staining image clearly reveals loss of brain structure at the stroke region (primary lesion) when compared to the contra-lesional hemisphere. 

### 3.1. Transmission Electron Microscope Studies to Define Ultrastructural Changes

Transmission electron microscope micrography’s reveal the ultrastructure of the contra-ipsi lesional hemispheres of the rat brain. The contra-lesional gray matter image show myelinated axons and unmyelinated neurons with normal features and integrated myelin sheath layer (concentric) surrounds the axonal neuron (Figure 1B_a_). The contra-lesional neurons looks intact in their structures with fully integrity. For the injured brain, sections at the PIZ region (Figure 1B_b_) and WM (Figure 1B_c–d_) had significant morphological ultrastructural alteration and damaged neurons. The images reveal that the neurons experienced swelling, forming multiple vacuoles and axonal degeneration (loss of myelin sheath membrane) (marked with black arrows).

### 3.2. Fourier Transform Infrared Imaging Spectroscopic Studies to Identify Biochemical and Molecular Changes

FTIR band assignments in this study were based on the specific spectral bands as defined in Table 1 [5,43]. We observed that spectral bands corresponding to lipid, protein, ester, nucleic acids and carbohydrates dominate the FTIR spectra. All samples were prepared, treated and processed in the same manner. In this study, we exploited for the first time to our knowledge, the approach of IR bio-spectroscopy to study the effect of the stroke on the rat brain after nine weeks. We investigated IR absorption in the range of 1500–1700 cm^−1^, which indicates the total protein region, is characteristic of amide band I and II of proteins. Amide I band mainly arises from backbone C=O stretching vibrations in the spectral range of 1700–1600 cm^−1^ [5,44]. Amide I band is composed of many contributions assigned to β-sheet within 1635–1610 cm^−1^; random coil at 1645–1630 cm^−1^ and α-helical at 1660–1650 cm^−1^. Amide II band arises from backbone of N-H bending and C=O stretching vibrations in the spectral range of 1580–1510 cm^−1^. We also investigated IR absorption in the range of 3100–2800 cm^−1^ that is the total lipid (C-H) region. Lipid component bands in this rang arise at 2955 cm^−1^, 2922 cm^−1^and 3012 cm^−1^ are assigned to the lipid methyl (CH_3_), lipid acyl (CH_2_) and olefinic = CH (unsaturated lipid) contents respectively were also studied [5,23,43]. The band arise at 1740 cm^−1^ is assigned for the lipid ester (phospholipid band) (C=O).

**Figure 2:**
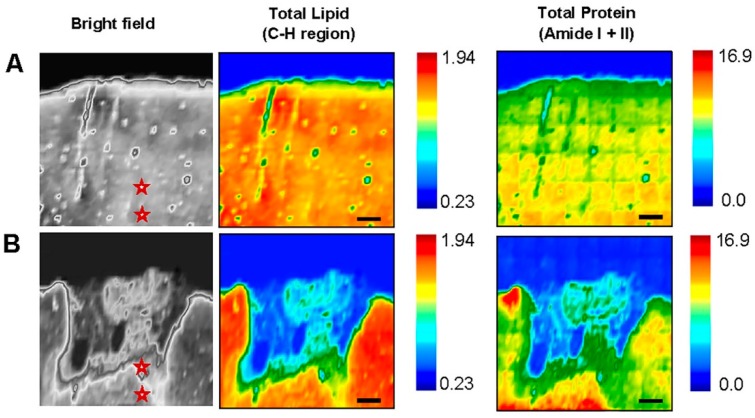
Fourier Transform Infrared (FTIR) biochemical images of Total Lipid (C-H region) and Total Protein (Amide I + II) on (**A**) Contra-lesional and (**B**) Ipsi-lesional hemisphere of nine weeks post stroke brain tissues. The images show significant changes in distribution of lipid and protein at peri-infract zone (PIZ) and white matter (WM). The stars in the bright field images shows where the spectra where taken. The color bars showing the intensity of the biochemical components—red as maximum and blue as minimum. Scale bar = 100 µm.

FTIR spectral maps were analyzed by comparing the lesioned (ipsilateral side) to the unlesioned (contralateral side) hemisphere since both hemispheres were treated in the same way (Figure 2A,B). The stars in the bright field images of the same regions indicates the location of the spectra derived from. FTIR spectroscopic analyses for the ROIs (ipsi-lesional PIZ and WM) identified the biochemical distribution of total lipid (TL) and total protein (TP). Lipid and protein contents were obtained from normalized area under the curve of C-H region (2800–3100 cm^−1^) and amide I + amide II (1500–1700 cm^−1^), respectively. The FTIR images clearly show reduction in the intensity of TL and TP in both ROI’s at ipsi-lesional side compared to contra-lesional region. This is attributed to the stroke induced damage to the ipsi-lesional PIZ and WM regions because these effects were not observed in the contralesional hemisphere. The color bars showing the intensity of the biochemical components—red as maximum and blue as minimum.

**Figure 3:**
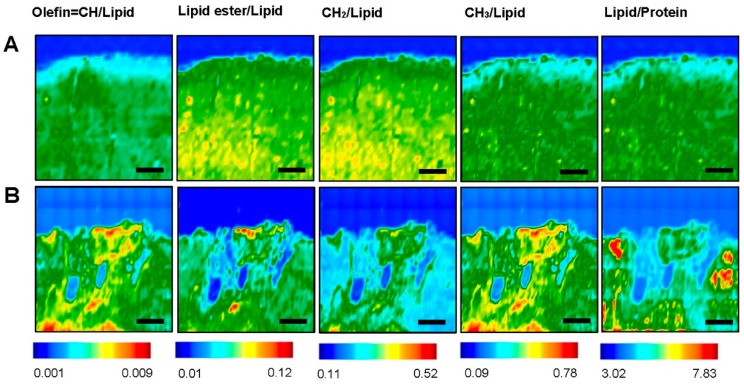
Fourier Transform Infrared (FTIR) images of different lipid components—Olefin=CH/Lipid, Lipid ester/Lipid, CH_2_/Lipid, CH_3_/Lipid and Lipid/Protein at (**A**) Contra-lesional and (**B**) Ipsi-lesional of nine weeks post stroke brain tissue. The color bars showing the intensity of the biochemical components—red as maximum and blue as minimum. Scale bar = 100 µm.

In order to understand and analyses the lipid biochemical alterations, the ratios of specific lipid bands to the total lipid (C–H stretching region) such as olefin=CH/Lipid, lipid ester/lipid, CH_3_/lipid and CH_2_/lipid have been investigated (Figure 3A,B). The unsaturation level of the lipid was measured using the olefinic = CH/lipid ratio. The lipid ester C=O/lipid ratio gives information about lipid ester concentration in lipids. Lipid acyl CH_2_/lipid ratio monitors the lipid chain length. CH_3_/lipid ratio gives information about methyl concentrations in the system.

**Figure 4:**
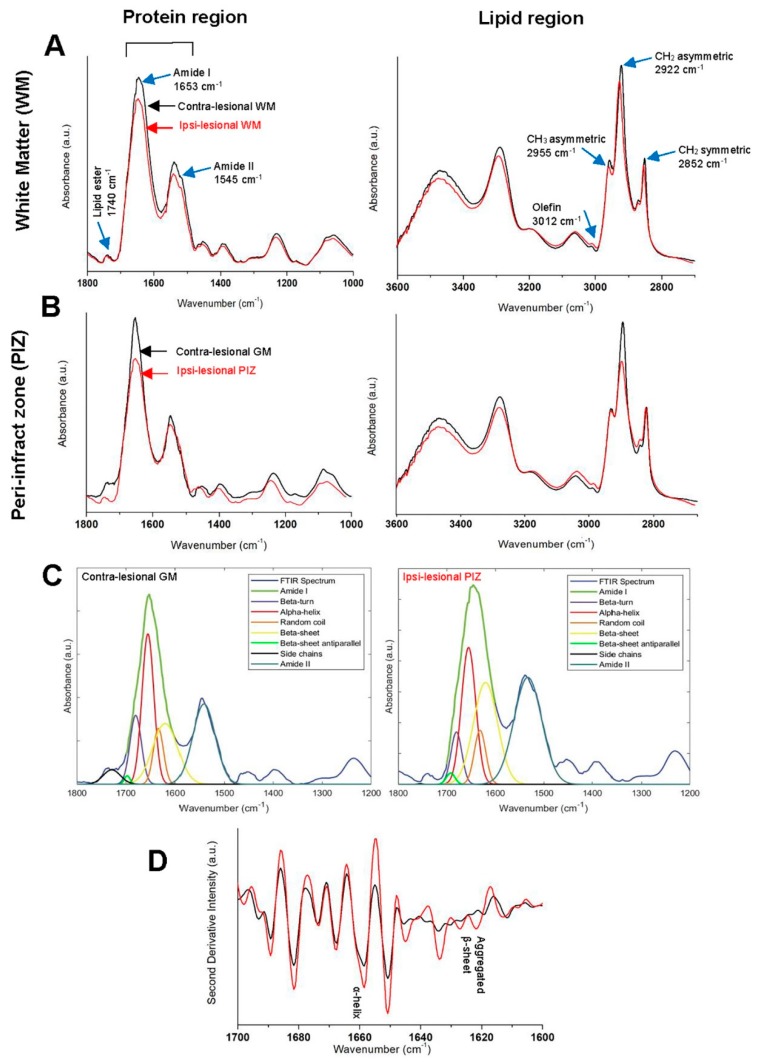
Comparison of Fourier Transform Infrared (FTIR) spectra from (**A**) White matter—WM and (**B**) Peri-infract zone—PIZ at contra-lesional (black) and ipsi-lesional (red) hemisphere of nine weeks post stroke showing the changes of peaks in Protein and Lipid regions separately. (**C**) Representative curve fitting spectra from– contra-lesional gray matter (GM) (left) and ipsi-lesional PIZ (right) quantifying changes in relative amount of aggregated protein from α-helix and β-sheet variations. (**D**) Representative second derivative spectra from PIZ showing α-helix and β-sheet variations. The peak assignments (name + wavenumbers) are specified by blue arrow.

We have also investigated and analyzed the lipid/protein ratio post stroke in the ROIs. This ratio provides information about membrane symmetry and function as well as its structure stability and integrity [43]. Homogenous distribution is observed on the contra-lesional side at cortex and WM (Figure 1A) whereas the ipsi-lesional side experienced significant changes in all the bio-chemical components ratios ( Figure 2A,B and Figure 3A,B). The results show that there is an elevation in the intensity of olefin=CH/lipid and CH_3_/lipid ratio at ipsi-lesional PIZ and WM whereas the lipid ester/lipid and CH_2_/lipid ratios showed drop in their intensity. More detailed comparison of WM and PIZ spectra are represented in Figure 4A–D respectively with changes in spectral curve fitting and second derivatives. The changes in the concentration values in total lipid, total protein, olefin/lipid, lipid ester/lipid, CH_2_/lipid, CH_3_/lipid and lipid/protein ratios are described below in Table 2.

#### 3.2.1. Contra-Lesional WM vs Ipsi-Lesional WM

The results showed that the ipsi-lesional WM is affected by the ischemic stroke insult. The normalized averaged FTIR spectra collected from the contra-lesional and ipsi-lesional WM regions are presented in Figure 4A. The ipsi-lesional WM spectrum revealed reduction in total lipid (TL) (0.93 ± 0.052 AU) and total protein (TP) (8.995 ± 0.0014 AU) in comparison to the contra-lesional hemisphere TL (1.39 ± 0.072 AU) and TP (9.061 ± 0.0014 AU), respectively. The results show that lipid structure and content at the ipsi-lesional WM has been affected. These changes are represented as the following: (a) considerable decline in lipid acyl chain length (CH_2_/lipid) ratio at ipsi-lesional WM and given by (0.125 ± 0.0001 AU) in comparison to contra-lesional WM where the ratios were higher (0.341 ± 0.0002 AU); (b) CH_3_/lipid ratio (0. 399 ± 0.0006 AU) increased in ipsi-lesional WM in comparison to contra-lesional WM, which exhibit lower ratio (0. 281 ± 0.0002 AU); (c) The spectral data reveal that there is a subtle decrease in the lipid ester content (lipid ester/lipid) at ipsi-lesional WM and given by (0.067 ± 0.0033 AU) in comparison to contra-lesional WM where the ratios were greater (0.086 ± 0.0031 AU) and (d) olefin/lipid ratio (level of unsaturation) at the ipsi-lesional WM is given by (0.0062 ± 0.0003 AU) raised on the affected hemisphere than the contra-lesional side (0.0045 ± 0.0003 AU). The results also showed that lipid/protein ratio exhibited significant dissimilar pattern between ipsi-lesional (6.367 ± 0.0013 AU) and contra-lesional WMs (6.991 ± 0.0018 AU). (*p* = 0.00023, n = 6).

#### 3.2.2. Contra-Lesional GM vs Ipsi-Lesional PIZ

The results revealed that ipsi-lesional PIZ region is greatly affected post ischemic stroke. Figure 4B shows the FTIR spectra obtained from contra-lesional cortex (GM) and ipsi-lesional PIZ. The results reveal that the PIZ region undergoes significant reduction in TL (0.59 ± 0.032 AU) and TP (11.003 ± 0.012 AU) comparing to the contra-lesional hemisphere TL (0.69 ± 0.022 AU) and TP (14.881 ± 0.043 AU), respectively. The results indicated that the PIZ lipid structure also has been altered since there is reduction in CH_2_/lipid (0.194 ± 0.0002 AU) is in comparison to contra-lesional GM, which exhibit higher ratio (0.233 ± 0.0001 AU). Consequently, CH_3_/lipid ratio increased in PIZ (0.394 ± 0.0007 AU) in comparison to contra-lesional GM, which exhibit lower ratio (0.165 ± 0.0003 AU). The results also show that lipid ester/lipid content is plunged and given by (0.057 ± 0.0062 AU) in comparison to contra-lesional cortex where the ratios were higher (0.063 ± 0.0051 AU). The results revealed that the unsaturated olefin/lipid (0.0052 ± 0.0005 AU) increased on the PIZ of the affected hemisphere than the unaffected side (0.0032 ± 0.0003 AU). Lipid/protein ratio showed significant dissimilarity between PIZ at ipsi-lesional region (5.256 ± 0.0033 AU) and unaffected contralateral side (5.887 ± 0.0013 AU). (*p* = 0.00039, n = 6). It is quite interesting to observe that PIZ region experiences significant structural changes in protein.

Second-derivative intensity at 1625 cm^−1^ determines the relative distribution of aggregated protein [45]. The relative level of aggregated protein content was identified from second-derivative intensity spectra and quantified from curve fitting the original spectra [22,45]. The results revealed that there is α-helix and random coil structures shift to the β-sheet conformation in the ipsi-lesional PIZ region. This increase in β-sheet content is associated with a reduction of the α-helix and random coil structures indicated by curve fitting spectra and second derivatives (Figure 4C,D). The changes in α-helix/Amide I, random coil/amide I and β-sheet/Amide I values, indicate biochemical alterations of protein post stroke in the PIZ region. On the contra-lesional gray matter, α-helix/Amide I was 51% and β-sheet/Amide I was 37%, which significantly changed to 32% and 66% accordingly in the PIZ (Table 3). In addition, the random coil played a role in forming more β-sheet in PIZ that can be agreed by its considerable drop from 12% to 10%. These results showed that there is a significant increase in the β-sheet conformation and resulting in protein aggregation at PIZ region of the brain. These protein biochemical changes was not observed in the ipsi-lesional WM.

We compared the values of the contra-lesional and ipsi-lesional hemispheres on the same sections would be critical in order to have better understanding of the attributed damages associated with induced stroke. The intensity values of TL, TP, lipid/protein, CH_2_/lipid, CH_3_/lipid, olefin/lipid and lipid ester/lipid mentioned above are tabulated in Table 2 and statistical analysis has been performed to see the significance of alterations took place in both ipsi-lesional PIZ and WM regions. These intensity or absorbance shows their arbitrary values and indicated by AU above.

## 4. Discussion

In this proposed study, we have applied a high-resolution approach as bio-diagnostics tool in order to examine the molecular alterations and tissue ultrastructure abnormalities in rat brain tissue sections of nine weeks post photothrombotic stroke. The applied techniques in this study are FTIR to study the bio-chemical and molecular changes and TEM to reveal the brain ultrastructure alterations. FTIR and TEM results emphasized that the ipsi-lesional PIZ and WM are significantly affected in our ischemic stroke model but at different degree which are in line with our previous studies [5]. Characterization the bio-chemical makeup changes in lipid, protein and lipid ester of ipsi-lesional PIZ and WM regions is essential in understanding significant information about the initiation and progression of neurodegeneration. Excitotoxicity, oxidative stress, inflammation and cell death are the main contributory pathways underlying stroke lesion progression and results in permanent or reversible neurological deficits. The stroke chronic effects lead to an imbalance in the production of harmful reactive oxygen species (ROS) over endogenous antioxidant protection strategies [46].

TEM showed that there is stretching, thinning, rupture and decreased in the myelin sheath thickness of nine weeks post ischemic stroke, which is consistent with increase the myelin sheath fragmentation. Electron microscopic studies of the ischemic brain revealed hypertrophy of the endothelial cells, increased mitochondria in sub endothelial space and narrowing of the capillary lumens which is showed in previous studies [47,48]. It also shows a middle cerebral artery after being exposed to ischemia. There was a significant increase in vesicle formation that occurred with the majority of vesicles located within in the ipsi-lesional PIZ and WM sections. The TEM image reveals that there are significant pathological and morphological ultrastructural alterations in cortical neurons post stroke. Chronic ischemic stroke leads to axonal degeneration (loss of myelin sheath membrane) and necrotic neurons. The results indicate that ischemic stroke can affect myelin sheaths and can cause to myelin disturbances in the cortical and ipsi-lesional WM neurons. 

The amount of lipids-proteins symmetry is an important factor affecting the membrane structure and dynamics [49,50]. FTIR spectra of lipids of the contra and ipsi-lesional hemispheres were analyzed. The significant alteration in the lipid acyl (CH_2_), methyl group (CH_3_), lipid ester (C=O) and olefinic =CH in the ipsi-lesional hemispheres of the insulted brain sections were found in this study. An increase in the methyl group CH_3_/ lipid ratio associated with reduction in the CH_2_/lipid and lipid ester content (C=O) might indicate the deterioration of lipids into short chain fragments by oxidative stress. Oxidative stress and resulting lipid peroxidation are responsible for the alteration of sub-cellular macromolecules [51,52,53,54,55,56,57,58]. Formation of degraded products such as lipid aldehydes, alkanes and alkyl radicals along with increased methyl CH_3_ concentration is influenced by lipid peroxidation [5,55,59]. Our results also show an increase in the olefinic =CH contents in the ipsi-lesional PIZ and WM regions, suggesting that insulted brain might experience oxidative stress post ischemic stroke. Increase olefinic=CH/lipid ratio in the ipsi-lesional PIZ and WM in comparison to the contra-lesional side indicates that the ischemic stroke could induce formation high concentration of unsaturated fatty acids and other by-products that contain higher concentration of double bonds around the insulted brain regions which can be a consequence to the lipid peroxidation process [5]. Our study showed the decrease in lipid acyl (CH_2_) content which leads to more double bond formation and increase in CH_3_ or methyl concentration. The results indicated that ischemic stroke causes oxidative degradation process of lipids cell membrane by a free radical chain reaction mechanism and results in brain tissue damage [60,61,62].

A precise lipid/protein ratio is calculated by the areas of the bands arising from lipids and proteins. Results indicated that this ratio decreases in the ipsi-lesional hemisphere for both PIZ and WM regions in comparison to the contra-lesional hemisphere. The reduction in the ratio suggests a decrease in the lipid content or an increase in the protein content or both [63]. In our study, there is a reduction in lipid and protein contents in the ipsi-lesional hemisphere within the PIZ and WM regions; the lipid/protein ratio was different within these regions relative to the contralateral hemisphere. The significant difference in lipid/protein ratio is consistent with chemical alterations caused by oxidative lipid and results in lipid peroxidation. Ischemic stroke resulting in imbalance of neurotransmitter release especially glutamate, the main excitotoxic neurotransmitter leading to influx of calcium, which triggers phospholipases and proteases that degrade essential membranes and proteins [64,65]. Furthermore, high calcium level stimulates oxygen radical production that directly damage sub-cellular biochemical structures [6]. Furthermore, free radical species has the potential in altering the endogenous functions of proteins, which may be neuroprotective [64,66]. Recent studies showed that less successful clinical recovery is correlated with increased peroxide concentration [67,68]. Ultimately, this cascades effect will lead to neuronal death through apoptosis, necrosis and autophagy [69].

Protein exposure to oxidative stress results in protein crosslinking, aggregation, fragmentation and denaturation, resulting in loss of function. The levels of the aggregated protein in the ipsi-lesional hemisphere was investigated from the amide I band at 1700–1600 cm^−1^ curve fitting. Interestingly, in the ipsi-lesional PIZ region, we observed that there is an increase in the absorption band of β-sheets at ≈1625–1635 cm^−1^ and associated with a decrease of α-helical absorption band at ≈1650–1660 cm^−1^ indicating protein aggregation and/or protein malfunction. This protein conformational change was not detected on the ipsi-lesional WM, indicated that the enrich lipid environment of the WM was able to protect the white matter protein content from the chemical alterations induced by ischemic stroke. Furthermore, the main bio-chemical alterations associated with chronic ischemic stroke is due to lipid oxidative stress and leads to lipid peroxidation. These results could not be obtained by other conventional biochemical assays [22,23]. The current techniques that could be used to determine the oxidative stress are exceptionally difficult and inaccurate [5,23]. Therefore, it is the ability of FTIR imaging bio-spectroscopic approach to our knowledge that used in this investigation is capable of spatially localize markers of oxidative stress in the PIZ, revealing this level of biochemical details and sensitive enough to detect these molecular changes associated with oxidative stress [5,22,70,71].

## 5. Conclusions

Our findings suggest that chronic ischemic stroke might cause a chemical oxidative stress damage to the ipsi-lesional PIZ and WM around the insulted brain region (primary lesion). This damage could cause bio-molecular and sub-cellular alterations of the brain tissues and results in affecting its neural integrity, structure and functionality. FTIR spectroscopy revealed that the bio-chemical and molecular alterations in the insulted ipsi-lesional hemisphere (PIZ and WM) associated with this stress are: (a) lipid peroxidation associated with oxidative stress and (b) change in the phospholipid content and (c) a shift from random coil and α-helical structures to β-sheet conformation in the PIZ region. These effects might damage the neural connection as well as the cell membrane and leads to neural deficits. These cascade effects could lead to neurodegeneration.

Moreover, from the above conclusions, it is clear that our results showed that FTIR bio-spectroscopy is a non-destructive, rapid and a refined technique to identify the biochemical and sub-cellular changes in insulted brain. It can provide information about lipid degradation and protein structural changes. Usually, detection of these changes requires biochemical methods that include sample homogenization and or treatment for immunoblotting or IHC, destroying the spatial-temporal integrity of the tissue samples and are not sensitive enough to detect these biochemical, molecular and sub-cellular alterations. Therefore, we conclude that FTIR can complement and expedite research into stroke and offer new approaches for neurodegenerative disorders. Antioxidant therapy reduces the extent of injury resulting from cerebral ischemia and enhances stroke recovery showed varying degrees of success. This approach inhibits ROS production or increase scavenging or degradation of ROS. However, early clinical studies in humans with anti-oxidation products have failed to translate success from bench to bedside. Our results suggest that a good therapeutic strategy should include administration of antioxidants as a key contributory element that provides protective effect from brain neurotoxicity. 

## Figures and Tables

**Table 1:** Spectral regions used for analytical interpretation for Stroke study.

	Infrared Band Assignment	Spectral Range (cm^−1^)	Comments
	Amide I + II	1700–1500	Total protein region
Protein components	Amide I	1700–1600	Specifically sensitive to protein secondary structure
	Amide II	1555–1535	Mainly ν(C–N) associated with proteins
	β-sheet	1610–1635	Functional protein, protein secondary structure
	α-helix	1660–1650	Functional protein, protein secondary structure
	Random coil	1645–1630	protein secondary structure
	C-H region	3100–2800	Total lipid region
Lipid component	ν_s_CH_2_	2852–2800	to measure lipid acyl chain length
	ν_as_CH_2_	2915–2930	
	ν_as_CH_3_	2950–2960	to measure concentration of methyl content
	*ν* (C = O)	1755–1715	to measure oxidative stress
	*ν* (olefinic = CH)	3000–3027	to measure unsaturated lipid

ν = stretching vibration; ν_as_ = asymmetric stretch; ν_s_ = symmetric stretch. * Resolutions Pro data analyzing software (V5.0), Cytospec (V2.00.03) Origin Lab (2019), Matlab (V2018b) and Cytospec (2.00.06) were used intensively to process all spectra and spectral images.

**Table 2:** Normalized values of biochemical components in regions of interest (ROI) at both sides.

	ROI	Total Lipid	Total Protein	Lipid/Protein	CH_2_/Lipid	CH_3_/Lipid	Olefin/Lipid	Lipid Ester/Lipid
Contra-lesional	GM	0.69 ± 0.022 *	14.881 ± 0.043 *	5.887 ± 0.0013 **	0.233 ± 0.0001 **	0.165 ± 0.0003 **	0.0032 ± 0.0003 **	0.063 ± 0.0051 **
	WM	1.39 ± 0.072 *	9.061 ± 0.0014 *	6.991 ± 0.0013 **	0.341 ± 0.0002 **	0.281 ± 0.0002 **	0.0045 ± 0.0003 **	0.086 ± 0.0031 **
Ipsi-lesional	PIZ	0.59 ± 0.032 **	11.003 ± 0.012 **	5.256 ± 0.0033 **	0.194 ± 0.0002 **	0.394 ± 0.0003 **	0.0052 ± 0.0005 **	0.057 ± 0.0062 **
	WM	0.93 ± 0.052 **	8.995 ± 0.0014 **	6.367 ± 0.0013 **	0.125 ± 0.0001 **	0.399 ± 0.0006 **	0.0062 ± 0.0003 **	0.067 ± 0.0033 **

The values are shown as ‘mean ± standard deviation’ for each group. The degree of significance is denoted as: * p < 0.05, ** p < 0.0001 and obtained by comparing each treated group with the control group. ROI: regions of interest; GM: gray matter; WM: white matter; PIZ: peri-infract zone.

**Table 3:** Ratios of protein alterations in GM and PIZ.

	Location of Brain	Contra-Lesional GM	Ipsi-Lesional PIZ
Ratios			
	α-helix/Amide I	51% *	32% *
	β-sheet/Amide I	37% *	66% *
	Random coil/Amide I	12% *	10% *

The degree of significance is denoted as: * p < 0.05. PIZ: peri-infract zone.
